# Nevus Sebaceous of Jadassohn With Secondary Cystic Papillary Hidradenoma

**DOI:** 10.7759/cureus.92156

**Published:** 2025-09-12

**Authors:** Jordan A Book, Rosemary Prejean, Jonathan M Joseph, Nicholas Culotta, Christopher Haas

**Affiliations:** 1 School of Medicine, Louisiana State University Health Sciences Center New Orleans, New Orleans, USA; 2 Dermatology, Louisiana State University Health Sciences Center New Orleans, New Orleans, USA

**Keywords:** benign tumor, ectopic glands, neoplastic transformation, nevus sebaceous, papillary hidradenoma

## Abstract

Nevus sebaceous of Jadassohn (NS) is a congenital cutaneous hamartoma that typically presents at birth as a plaque on the face, scalp, or neck. While NS is associated with a risk of neoplastic transformation, the majority of secondary tumors are benign, with the most common being trichoblastomas and syringocystadenomas. In this report, we present a rare case of a 63-year-old male with a right parietal scalp NS with a secondary cystic papillary hidradenoma (HP), which is a benign tumor that almost exclusively occurs in the perineal region. Histopathologic examination was consistent with NS, revealing hyperkeratosis, parakeratosis, increased sebaceous glands, ectopic apocrine glands, and anomalous duct sweat gland hyperplasia. The lesion also demonstrated characteristics of HP, a neoplasm that has not been well-documented in association with NS. This case expands understanding of neoplasms that arise within NS and highlights histologic characteristics that differentiate HP from other neoplasms.

## Introduction

Nevus sebaceous of Jadassohn (NS), or organoid nevus, is a congenital cutaneous hamartoma that occurs in approximately 0.1-0.3% of individuals [1]. Males and females are affected equally, and there is no apparent ethnic predominance [2, 3]. NS typically presents at birth as a partially or completely alopecic plaque on the scalp (57.1%), face (33.8%), or neck (8.3%) [4]. It may vary in color, commonly appearing skin-colored, yellow-orange, or brown-black [2]. The lesion may be isolated or a component of an epidermal nevus syndrome, with the most well-known subtype being Schimmelpenning syndrome. This syndrome is characterized by linear NS along Blaschko lines, central nervous system abnormalities, ocular abnormalities, and seizures [5].

During puberty, benign enlargement of NS occurs due to hormone-driven hyperplasia of sebaceous glands and adnexal structures. With age, the surface becomes more verrucous, along with an increase in acanthosis, hyperkeratosis, hyperplastic sebaceous glands, and ectopic apocrine glands [4]. During infancy, the differential diagnosis includes aplasia cutis congenita, epidermal nevus, and mastocytoma [6]. In adulthood, it includes seborrheic keratosis, verruca vulgaris, and sebaceous hyperplasia [7, 8]. Diagnosis is confirmed based on clinical and histopathologic findings [9]. Treatment remains controversial, with suggestions ranging from pre-pubertal excision to observation. Ultimately, management is individualized based on patient history, cosmetic concerns, symptoms such as pruritus, and the potential risk of secondary neoplasms. On the other hand, if a malignant lesion develops within NS, complete excision of the nevus is recommended [10].

Though the majority of NS remain unproblematic, about 10-20% of lesions give rise to secondary neoplasms, most of which are benign [2, 3, 11]. A 2024 meta-analysis found that secondary neoplasms within NS occurred at a rate of 12.8%, with trichoblastomas and syringocystadenomas being the most prevalent [11]. Trichoblastomas arise from the proliferation of follicular germ cells and typically present as skin-colored papules. Syringocystadenomas originate from apocrine and eccrine sweat glands, typically presenting as isolated patches or nodules with possible vesicles and exudate [2]. Basal cell carcinoma (BCC) is the most common malignant neoplasm associated with NS and was historically thought to occur at an incidence as high as 22.2% [12]. However, recent studies suggest many of the lesions previously diagnosed as BCC were misclassified trichoblastomas, and that the true incidence lies below 2% [3, 4, 13, 14].

Papillary hidradenoma, also known as hidradenoma papilliferum (HP), originates from the secretory portion of anogenital mammary-like and apocrine glands. The lesion arises almost exclusively in the perineal region; however, ectopic HP can develop outside of this region, most often on the head or neck [13]. HP typically presents as a firm, red, dermal or subcutaneous nodule without connection to the epidermis [14, 15]. It predominantly occurs in females aged 30-49 years old, though ectopic HP is more common in males [13, 15]. Although malignant transformation is extremely rare, treatment is complete excision [14, 15]. To our knowledge, the occurrence of HP within NS has not been well-documented in published literature. In this report, we present a rare case of NS with a secondary cystic papillary hidradenoma on the scalp, expanding the understanding of neoplasms associated with this congenital lesion.

## Case presentation

**Figure 1:**
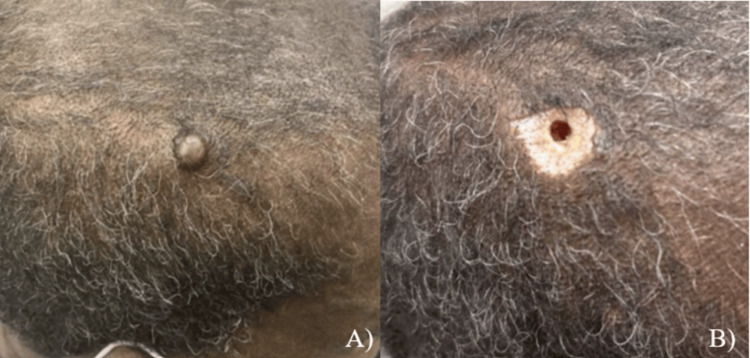
Clinical images of skin lesion on the right parietal scalp. (A) Initial presentation of 1 cm nodular growth overlying a hyperpigmented, verrucous plaque and (B) skin findings after shave excision of the nodule.

A 63-year-old African American male with a history of Human Immunodeficiency Virus (HIV), well-controlled on Highly Active Antiretroviral Therapy (HAART), hypertension, hyperlipidemia, and end-stage renal disease (ESRD) presented to the clinic for evaluation of an enlarging nodule on the right side of his scalp (Figure 1A). He reported having a thin, raised plaque in the area since childhood and noted a gradual growth of this nodule within the plaque over the preceding two years. During this time, the patient had no other dermatologic evaluations. He denied pain, pruritus, drainage, or any other accompanying symptoms associated with the lesion. He also denied any personal or family history of similar lesions.

Physical examination revealed a 1 cm, skin-colored, smooth nodule on the right temporoparietal region overlying a 2.1 cm x 1.7 cm hyperpigmented, verrucous, alopecic plaque. A shave biopsy was performed as it is a standard, minimally invasive practice for sampling superficial and dermal lesions. The specimen adequately extended to the mid-dermis to capture both apocrine and eccrine components, while minimizing patient discomfort and scarring. Following shave removal, a tunnel-like opening remained, extending from the dermis into the subcutaneous tissue (Figure 1B).

**Figure 2:**
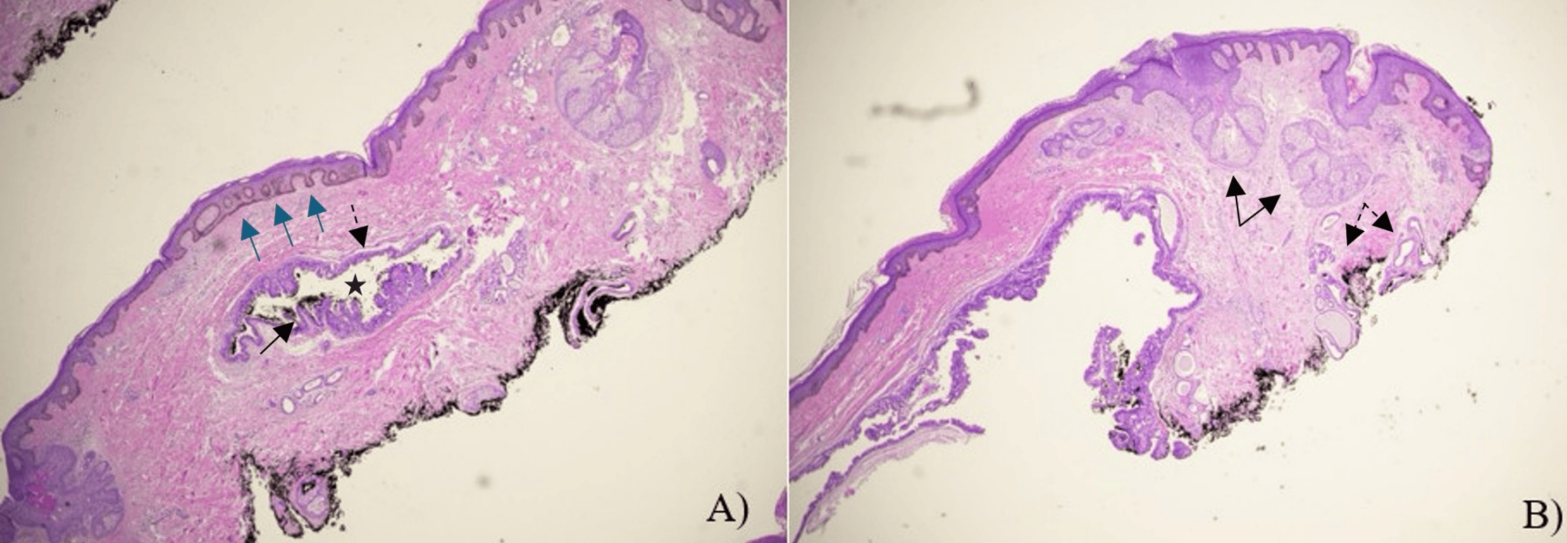
Low magnification histologic images of the specimen using hematoxylin and eosin (H&E) staining. Figure 2A demonstrates a well-circumscribed lesion with dermal cystic eosinophilic space (black star), papillary projections (black solid arrow), and a double-lined epithelium (black dashed arrow), which are features consistent with papillary hidradenoma (HP). It also shows histologic characteristics of nevus sebaceous (NS) such as acanthosis, papillomatosis, and basal layer hyperpigmentation (blue solid arrows point to section encompassing all three findings). Figure 2B further exhibits findings consistent with nevus sebaceous, with prominent acanthosis and papillomatosis, increased sebaceous glands (black solid arrows), and apocrine glands (black dashed arrow).

Biopsy of the lesion was notable for papillomatosis, hyperkeratosis, and increased basal layer pigmentation. There were increased sebaceous glands, ectopic apocrine glands, and anomalous duct sweat gland hyperplasia. These findings were consistent with NS [4]. Additionally, the biopsy revealed dermal cystic eosinophilic spaces, papillary projections, and a double-lined epithelium, which are features consistent with HP (Figures 2A-2B) [16]. Features of eccrine, apocrine, and papillary hidradenoma were all present, giving the lesion a hybrid appearance. Immunohistochemical staining with S-100 and smooth muscle actin (SMA) demonstrated myoepithelial cells surrounding the tumor, supporting the diagnosis of a benign adnexal tumor consistent with HP. Carcinoembryonic antigen (CEA), epithelial membrane antigen (EMA), and cytokeratin 7 (CK7) staining were positive, highlighting hidradenoma cells and supporting apocrine differentiation. Clinical and histological evaluation aligned with a diagnosis of NS with cystic papillary hidradenoma. The patient was offered total excision of the NS to decrease the risk of recurrence or additional neoplastic transformation. However, the patient chose to proceed with clinical monitoring.

## Discussion

NS is a congenital cutaneous hamartoma of the pilosebaceous follicular unit, epidermis, and other adnexal structures [2]. Lesion development has been linked to post-zygotic mutations in the Ras protein family, most often *HRAS *and *KRAS*, which are suggested to predispose them to secondary neoplastic growth, both benign and malignant [2, 17]. In one study that analyzed tissue from 65 NS lesions, 97% had a mutation in *HRAS *and/or *KRAS *[17]. In another study that sequenced 31 NS samples, 89% had a mutation in *HRAS *and/or *KRAS*. Additionally, immunohistochemistry (IHC) with phosphorylated extracellular signal-regulated kinase (pERK) staining showed increased expression in NS samples compared to normal epidermis. This indicates elevated mitogen-activated protein kinase (MAPK) pathway activity, which is consistent with *RAS *hyperactivation [18].

In contrast to normal pubertal change, growth of NS in adulthood raises concerns for the development of an associated secondary neoplasm, as seen with this patient [4]. The secondary neoplasm detected in this case was consistent with HP. This lesion is primarily found in the anogenital region, but it rarely occurs in ectopic areas such as the breasts, head, and neck [13]. Multiple case reports have noted ectopic HP on the scalp, though to our knowledge it has not been reported in association with NS [19, 20].

Histologically, these tumors exhibit cystic eosinophilic spaces with papillary and adenomatous structures lined by a double layer of epithelium. The basal layer is composed of cuboidal cells, while the luminal layer consists of columnar cells [16]. To rule out other diagnoses in this case, we noted that histological examination showed no follicular germ cells indicative of trichoblastoma, no basaloid cells suggestive of basal cell carcinoma (BCC), and no atypical mitotic figures or necrosis to suggest other malignancy [2]. Non-neoplastic causes were ruled out with negative bacterial and fungal stains, excluding infectious etiologies such as folliculitis or tinea.

There is no clear consensus on surveillance for conjoined NS-HP lesions due to the benign nature, low malignant potential, and low risk of HP recurrence after excision [10]. However, given NS's known risk for additional neoplasms, we proposed an ideal plan that includes an initial 6-month dermoscopy to monitor for recurrence or new nodules, followed by annual clinical examinations and dermoscopy. Biopsies should be considered for any nodules >5 mm or changes in color, texture, or size, aligning with general recommendations for NS surveillance to balance monitoring with patient burden [4]. Being an individual case description, this report provides limited insight into the prevalence of this secondary neoplasm within NS. Additionally, this patient's lack of follow-up limits information on the clinical outcome over time.

## Conclusions

This case describes a rare presentation of an ectopic HP within an NS lesion on the scalp, a region with relatively low apocrine gland density. It highlights an example of a benign neoplasm that may arise within NS and describes histologic characteristics that differentiate it from other common lesions. Due to the overall low risk of malignancy, management is individualized based on clinical features, histology, and patient preference, though surveillance should be considered to facilitate early detection of any atypical or malignant changes.

## References

[1] Alper J, Holmes LB, Mihm MC (1979). Birthmarks with serious medical significance: nevocellular nevi, sebaceous nevi, and multiple café au lait spots. J Pediatr.

[2] Neto MP, Assis BR, Andrade GR (2022). Sebaceous nevus of Jadassohn: review and clinical-surgical approach. An Bras Dermatol.

[3] Idriss MH, Elston DM (2014). Secondary neoplasms associated with nevus sebaceus of Jadassohn: a study of 707 cases. J Am Acad Dermatol.

[4] Kamyab-Hesari K, Seirafi H, Jahan S, Aghazadeh N, Hejazi P, Azizpour A, Goodarzi A (2016). Nevus sebaceus: a clinicopathological study of 168 cases and review of the literature. Int J Dermatol.

[5] Pandya M, Thool AR, Daigavane S (2024). Linear nevus sebaceous syndrome: clinical presentation and management considerations. Cureus.

[6] Lopez AS, Lam JM (2019). Nevus sebaceous. CMAJ.

[7] Zhou L, Fu Y, Huang J (2025). Characteristics and differential diagnosis of common verrucous proliferative skin diseases under dermoscopy and reflectance confocal microscopy. Zhong Nan Da Xue Xue Bao Yi Xue Ban.

[8] Sahu P, Lakra S, Dayal S (2020). Nevus sebaceous on face: histopathological and dermoscopic correlation. Indian Dermatol Online J.

[9] Asfar M, Styles A, Somach S (2023). "Sebaceous holes": a clue to the diagnosis of nevus sebaceus. J Cutan Pathol.

[10] Moody MN, Landau JM, Goldberg LH (2012). Nevus sebaceous revisited. Pediatr Dermatol.

[11] Pang S, Cevik J, Sreedharan S, Wilks DJ (2024). Rate of benign and malignant secondary tumors associated with nevus sebaceous: a systematic review and meta-analysis. Ann Plast Surg.

[12] Rosen H, Schmidt B, Lam HP, Meara JG, Labow BI (2009). Management of nevus sebaceous and the risk of Basal cell carcinoma: an 18-year review. Pediatr Dermatol.

[13] Chauhan H, Tandon P, Potlia I, Jain E (2020). Rare and unusual occurrence of ectopic hidradenoma papilliferum in maxillofacial region. J Oral Maxillofac Pathol.

[14] Kambil SM, Bhat RM, D'Souza DC (2014). Hidradenoma papilliferum of the vulva. Indian Dermatol Online J.

[15] Patel S, Lambert WC, Behbanani S, Espinal-Mariotte JD, Lee P (2020). Hidradenoma papilliferum: everyone else's diagnosis. Indian J Dermatol.

[16] Shimon SV, Alenezi SM, Camela E, Maderal AD, Romanelli P (2024). A rare case of ectopic hidradenoma papilliferum of the external auditory canal. Cureus.

[17] Groesser L, Herschberger E, Ruetten A (2012). Postzygotic HRAS and KRAS mutations cause nevus sebaceous and Schimmelpenning syndrome. Nat Genet.

[18] Sun BK, Saggini A, Sarin KY, Kim J, Benjamin L, LeBoit PE, Khavari PA (2013). Mosaic activating RAS mutations in nevus sebaceus and nevus sebaceus syndrome. J Invest Dermatol.

[19] Moon JW, Na CH, Kim HR, Shin BS (2009). Giant ectopic hidradenoma papilliferum on the scalp. J Dermatol.

[20] Küçükünal A, Esen K, Kıvanç Altunay İ, Aksu Cerman A (2015). Ectopic hidradenoma papilliferum on the scalp. J Turk Acad Dermatol.

